# Origin and Diversification of Dung Beetles in Madagascar

**DOI:** 10.3390/insects2020112

**Published:** 2011-04-20

**Authors:** Andreia Miraldo, Helena Wirta, Ilkka Hanski

**Affiliations:** Metapopulation Research Group, Department of Biosciences, University of Helsinki, P.O. Box 65, FI-00014, Finland; E-Mails: helena.wirta@gmail.com (H.W.); ilkka.hanski@helsinki.fi (I.H.)

**Keywords:** dung beetles, Madagascar, radiation, adaptation, phylogeography, speciation

## Abstract

Madagascar has a rich fauna of dung beetles (Scarabaeinae and Aphodiinae) with almost 300 species described to date. Like most other taxa in Madagascar, dung beetles exhibit an exceptionally high level of endemism (96% of the species). Here, we review the current knowledge of the origin and diversification of Malagasy dung beetles. Based on molecular phylogenies, the extant dung beetles originate from eight colonizations, of which four have given rise to extensive radiations. These radiations have occurred in wet forests, while the few extant species in the less successful radiations occur in open and semi-open habitats. We discuss the likely mechanisms of speciation and the ecological characteristics of the extant communities, emphasizing the role of adaptation along environmental gradients and allopatric speciation in generating the exceptionally high beta diversity in Malagasy dung beetles. Phylogeographic analyses of selected species reveal complex patterns with evidence for genetic introgression between old taxa. The introduction of cattle to Madagascar 1500 years ago created a new abundant resource, onto which a few species have shifted and thereby been able to greatly expand their geographical ranges.

## 1. Introduction

Madagascar has an exceptional biota characterized by a remarkably high level of endemism at all taxonomic levels [1]. Species level endemism reaches 100% in many taxa and there are many families and tribes that occur only in Madagascar. For example, all of the native amphibians and terrestrial mammals in Madagascar are endemic [2]. Because of the exceptional endemism and generally high species diversity, Madagascar is considered one of the most important biodiversity hotspots in the world [3].

Madagascar is the world's fourth largest island (578,000 km^2^) and its origin dates to the break-up of the supercontinent Gondwana 160–130 Mya. Following the break-up from the African continent, Madagascar was still connected to India, Antarctica and Australia for a long time, becoming completely isolated from these land masses 90–80 Mya [4–8]. Long physical isolation has undoubtedly contributed to the uniqueness of Madagascar's biota and promoted the differentiation of Gondwanan relic groups (e.g., [2,9–12]). However, most of the splits between Madagascan clades and their sisters are much younger than the Gondwanan geological events and therefore cannot be explained by Gondwanan vicariance. Based on dated phylogenies, many endemic lineages in Madagascar must be part of more recent radiations due to overseas dispersal (e.g., [13–15]).

In addition to the long history of isolation, the topographic relief of Madagascar is likely to have played an important role in radiations. Madagascar has a mountain chain running for 1300 km along its latitudinal axis. The mountain chain is associated with much variation in vegetation and climate not only along the latitude but especially along the longitude, as humidity precipitates in the eastern slopes of the mountain chain creating an east-west gradient in rainfall. Thus, the tropical wet regions in the northeast and east are very different from the sub-arid regions in the southwest, and the boundaries between bioclimatic regions are often very abrupt [2]. The presence of several isolated mountain massifs in northern (e.g., Marojejy and Montagne d'Ambre) and southern (e.g., Andohahela) regions of Madagascar, disconnected from the central ones, are also likely to have triggered species diversification, either by serving as mountain refugial regions during dryer climatic periods or by allowing adaptive speciation along the elevational gradients [1]. In fact, several different mechanisms of species diversification have been proposed for Madagascar [1,15], all of them intimately associated with the topographic and climatic particularities of the island. In brief, the large area and great physiographic complexity of Madagascar have provided a multitude of different climates and habitats that have allowed many lineages to diversify, following successful overseas colonization, through extensive adaptive and non-adaptive radiations.

**Figure A1:**
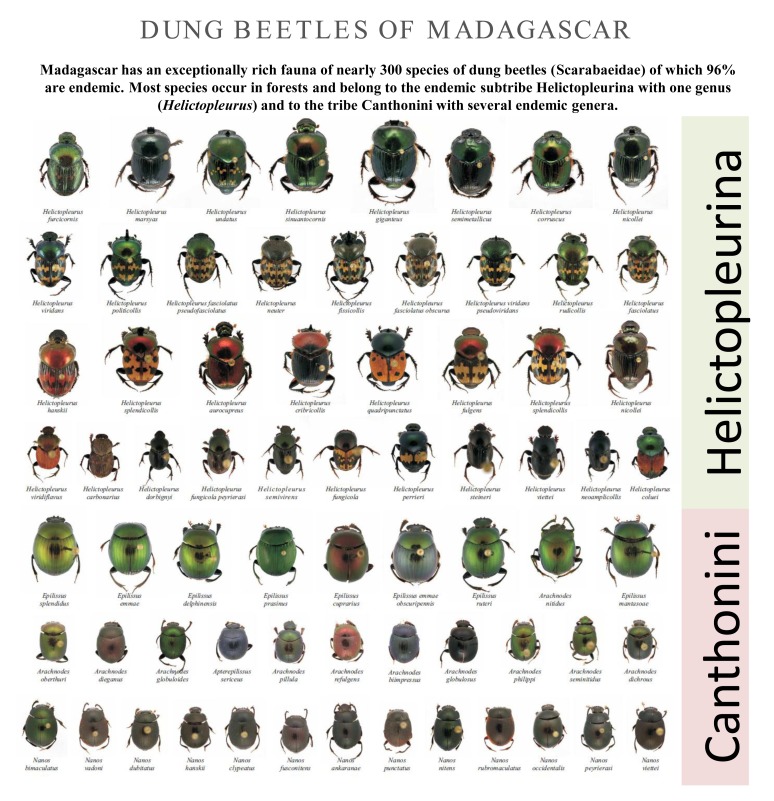
Dung beetles of Madagascar. Photographs of several Canthonini and Helictopleurina species endemic to Madagascar.

Dung beetles are a great example of Madagascar's biological uniqueness: of the almost 300 species described to date, 285 (96%) are endemic, with several endemic genera and 1 endemic subtribe (Helictopleurina in the tribe Oniticellini) (Figure A1). In recent years, knowledge of Malagasy dung beetles has increased greatly with the publication of taxonomic revisions [16–25], reconstruction of molecular phylogenies [24,26–28], as well as phylogeographic [29–31] and ecological studies [32–35]. Here we review and summarize the current knowledge on the ecology and evolutionary biology of dung beetles in Madagascar. We start by describing the composition and structure of Malagasy dung beetle communities, next we discuss their evolutionary origin and compare successful *vs*. unsuccessful radiations, and we conclude by discussing the likely ecological and evolutionary mechanisms that are responsible for the distributional patterns we observe today.

## 2. Dung Beetle Communities in Madagascar

Malagasy dung beetles belong to two sub-families, the numerically dominant Scarabaeinae, represented by approximately 270 species, and Aphodiinae with approximately 30 species. The Scarabaeinae consists almost entirely of endemic species and genera belonging to four tribes: Canthonini, Oniticellini (subtribe Helictopleurina), Scarabaeini and Onthophagini. Levels of endemism, divergence times, mechanisms of species diversification and the extent to which lineages have radiated vary greatly between the lineages (Table 1). The most diverse tribe is the old Gondwanan tribe Canthonini, with more than 190 described species, all endemic to Madagascar, followed by the endemic subtribe Helictopleurina with 66 extant taxa [16–23,25,36–41]. Scarabaeini includes three endemic species, while Onthophagini is represented by six species, of which four are endemic and two represent recent introductions [42].

The great majority of dung beetles in Madagascar are strictly forest inhabitants, mainly associated with the eastern tropical wet forests, while a small number of species occurs in the western dry open and semi-open areas. Forests are inhabited exclusively by endemic species in Canthonini and Helictopleurina, whereas open habitats have representatives of all the four tribes of Scarabaeinae as well as endemic and non-endemic Aphodiinae [27,32,33]. Local dung beetle communities in Madagascar differ in many ways from communities in other comparable tropical areas. One of the main differences is in local species richness: wet forest communities in Madagascar have clearly fewer species (around 30 species, [35]) than communities elsewhere in the tropics (more than 50 species, [44]). This cannot be explained by a limited size of the species pool, as the total number of species in Madagascar (300 species) is greater than the total number of species in other comparable large islands (Borneo 120 species and Sumatra 112 [45]; these numbers may be underestimates but the totals must be much less than 300). Instead, low species richness (alpha diversity) in local communities in Madagascar is likely to be due to the ecological conditions. In general, patterns in the geographical occurrence of dung beetles are intimately related to the occurrence of the major mammalian taxa [35,46,47], the main resource providers for dung beetles. As Madagascar lacks native large herbivorous mammals (ungulates), dung and carrion are produced by an exceptionally narrow range of species, mainly medium-sized lemurs, rodents and insectivores. Furthermore, the density and biomass of these mammal communities in Madagascar are relatively low, which leads to low resource availability to dung beetles [35].

Despite the limited diversity and availability of resources, Madagascar has an exceptionally diverse dung beetle fauna. The low alpha diversity is compensated for by an exceptionally high regional turnover in species composition [35]. High total species richness reflects the long evolutionary history over several tens of millions of years (discussed below), but ecological factors also play a role in community assembly. In a recent study, Viljanen *et al.* [33] compared the composition and structure of two wet forest dung beetle communities in south-east (Ranomafana) and in north-east (Masoala), and they observed that both communities have similar numbers of species but the species were almost completely different. Within each community, co-existing species showed significant ecological differences in resource use, body size and elevational occurrence. Importantly, in each community there were species with similar traits related to resource use, *i.e.*, different species fill the same ecological niche in different communities. These observations strongly indicate that interspecific interactions restrict the number of locally co-existing species [33–35]. Although not conclusive, these results also suggest that resource competition is responsible for the limited numbers of co-existing species in local communities.

## 3. Origin of Malagasy Dung Beetles

The extant dung beetle communities in Madagascar appear to have originated from at least eight independent colonizations (Table 1). These colonizations have given rise to an array of different lineages, from very diverse (Canthonini) to very species poor (Scarabaeini). Phylogenies using mitochondrial and nuclear genes suggest that colonizations have occurred during the Cenozoic [27,28]. However, it is worth emphasizing that all time estimates reported to date rely on sequence divergence rate estimates of mitochondrial COI gene of other insect taxa. The estimates are therefore very approximate, as the rates may differ greatly between taxa and may change across different geological times.

The most diverse tribe, Canthonini, includes three separate lineages: one consisting of the genera *Arachnodes*, *Epilissus* and *Apterepilissus* (the *Arachnodes* clade, sensu [28]), another lineage consisting of *Epactoides* (the *Epactoides* clade) and a third one consisting of the genera *Nanos* and *Apotolamprus* (the *Nanos* clade). The ancestors of these lineages arrived at Madagascar in at least three independent colonization events. The first to arrive was most likely the ancestor of the *Arachnodes* clade, as divergence within this clade appears to have started earlier (79–49 Mya) than reported for the other Malagasy dung beetles. *Arachnodes* radiation was followed by the one in *Epactoides*, with divergence within the group estimated to have started 30–19 Mya. The divergence of *Epactoides* from its closest relative *Ochicanthon* from India [28] occurred 38–24 Mya. These dates imply overseas colonization of Madagascar or India, as the two land masses lost connection about 80 Mya. The youngest Canthonini clade in Madagascar is the *Nanos* clade with divergence from the most recent common ancestor estimated to have occurred 24–15 Mya [28]. In the case of the endemic subtribe Helictopleurina, monophyly supports a single colonization event and therefore a single origin for the 66 extant taxa. Wirta *et al.* [27] estimated the time of divergence of Helictopleurina from its closest African relative (*Oniticellus planatus*) at 44–28 Mya, also supporting overseas colonization of Madagascar. Divergence within the subtribe Helictopleurina has occurred since 35–23 Mya. Within the less diverse tribes, there is one origin for two of the three endemic Scarabaeini species with the origin of diversification estimated at 24–15 Mya [43], and at least three independent colonizations for the six Onthophagini species [27], though their time of arrival and diversification in Madagascar is unknown. Finally, we reiterate once more that the above estimates are very approximate and further research may change our conclusions. The great uncertainty of the time estimates is highlighted by the work of Sole and Scholtz [48], who date the origin of African Canthonini at a much more recent time, around 40 Mya, than our estimate of colonization of Madagascar by the *Arachnodes* clade.

Due to its proximity to Madagascar, the African mainland is the likely source of the colonizations. The strongest evidence for an African origin comes from the phylogenetic relationships between Helictopleurina and its closest relative, which has been identified as an African Oniticellini [27]. For Canthonini, an Asian origin cannot be excluded especially for the *Epactoides* clade. Although the most recent phylogenetic study of the Malagasy Canthonini failed to resolve their relationships with other Scarabaeinae throughout the world, it identified a close relationship between the *Epactoides* clade and *Ochicanthon* from India [28]. This is in agreement with morphological analysis also reveals a close relationship between the Oriental *Ochicanthon* and the Malagasy *Epactoides* [24]. There is also morphological support for a close relationship between the *Nanos* clade and some New Caledonian taxa (*Pseudonthobium*) [19], though there is presently no genetic data to examine this relationship.

The origin of dung beetles in Madagascar is related to the wider debate about the origin of the dung beetle fauna in general and the biogeographic and evolutionary processes responsible for the global distribution of dung beetles. The basal tribes Canthonini and Dichotomiini appear to have evolved in Gondwana, supported by the presence of apparent relict taxa in Africa [47–51]. Based on the high generic and tribal endemism in the modern southern continents, including Madagascar, it has been hypothesized that vicariance following the break-up of Gondwana was responsible for the start of the diversification in many of the “old” dung beetle lineages [47]. Though intuitive, the generality of this biogeographic scenario is still a matter of debate (see [51] for a detailed review). The molecular phylogenies for Malagasy dung beetles are not consistent with the vicariance hypothesis [27,28,43]: vicariance from Gondwanan relicts would imply very old lineages in contrast to the dates presented above, which imply that most if not all Malagasy dung beetle lineages are younger and originate from overseas colonizations.

## 4. Successful *vs.* Unsuccessful Radiations

As described above, the taxonomic composition of Malagasy dung beetles is highly biased, with the vast majority of species belonging to the old Gondwanan tribe Canthonini and the endemic subtribe Helictopleurina. Of the eight independent colonization events, four have led to extensive radiations while four others have failed to radiate. The four successful lineages are the *Arachnodes* (101 species), *Epactoides* (37) and *Nanos* (61) clades in Canthonini and Helictopleurina (66 species) in Oniticellini. In contrast, both Scarabaeini and Onthophagini colonizations have resulted in only one or two endemic extant species. Why have some of the lineages experienced extensive radiations while others have failed to radiate at all? As we describe below, this is most likely related to the ecology and evolutionary biology of the lineages, but the order of colonizations may also have played an important role.

The four successful radiations differ in age, number of extant species and in the distribution of ecological traits. The two oldest and largest lineages, *Arachnodes* and Helictopleurina, exhibit the greatest extent of ecological differentiation and have a higher proportion of dung specialist species than the two other large clades. *Arachnodes* and Helictopleurina were the first to radiate and they seem to have evolved to use the most favorable resource available for dung beetles in Madagascar (primate dung). Although all the four large radiations occur primarily in wet forests, the two oldest radiations have also succeeded in occupying dry forests and some of the species have adapted to living in open areas, feeding currently on cattle dung (discussed below). It is also in the two oldest radiations that the largest species in body size exist, probably a result of evolution in association with the now extinct Malagasy megafauna. Resource competition between the first lineages to radiate has been reduced by the evolution of largely different diel activities, with *Arachnodes* being mainly nocturnal or crepuscular [33] and Helictopleurina diurnal [27]. Such nearly complete division of diel activity between the dominant lineages is not common in other tropical regions [52]. The success of the older radiations seems to be explained by the earlier arrival of their ancestors in Madagascar, which were faced with an array of novel ecological opportunities and habitats. The great success of the early arrivals may have influenced the subsequent radiations in the other lineages and contributed to the failure of others to radiate at all.

The two remaining successful radiations are very different from each other. *Epactoides* is mainly composed of small species that have diverged substantially in their ecologies. Although forming a very diverse group, *Epactoides* species are mostly uncommon. Several species in this group are wingless and associated with high elevations [24]. Ecological differentiation and adaptation into marginal ecological conditions seem to have been the key for the success of this clade. This leaves one more large radiation, the *Nanos* clade, which is the most recent one (Table 1). Being the latest lineage to radiate is likely to have imposed some restrictions on the evolution of the species in this group, as competition for resources must have been intense. Nevertheless, many species in the *Nanos* clade are abundant and ecologically successful, often even dominating numerically local dung beetle communities. The species are morphologically and ecologically similar, and they are mainly diet generalists, feeding on carrion and dung. A generalized diet may have been the key to the ecological success of this group. We return below to their phylogeographic relationships.

The species-poor Malagasy Onthophagini and Scarabaeini are in strong contrast with the above discussed species-rich lineages (Table 1). Onthophagini is by far the most species-rich and widespread tribe in the world, including roughly half of the total number of described Scarabaeinae species [51]. Although Onthophagini occur commonly in forests elsewhere in the tropics, they have failed to colonize forests in Madagascar, where they occur only in dry open areas [32]. A possible explanation is the success of Canthonini and Helictopleurina: these taxa appear to have occupied all the major dung beetle niches in forests [32]. It is particularly noteworthy that Helictopleurina are ecologically very similar to Onthophagini. Although a time-calibrated phylogeny for the Malagasy Onthophagini is not yet available, it is likely that they have arrived much later than Helictopleurina. Regarding Scarabaeini, although the closest species to Malagasy Scarabaeini, the African *Scarabaeus*, are very successful in Africa, this group has not thrived in Madagascar [43]. The distribution of *Scarabaeus* is centered primarily on drier regions in winter shrublands, dry savannas and upland grasslands [51]. In Madagascar, the three species of Scarabaeini are restricted to dry areas, occurring in the south and west of the island. It is likely that their failure to radiate is related to the limited resources for dung beetles in dry areas in Madagascar, as most large mammals have occurred in forests before the recent introduction of cattle [32,43].

## 5. Shifts in Resource Use in Ecological and Evolutionary Time Scales

The greatest number of Scarabaeinae species worldwide feed on the dung of large-bodied herbivores [51,52]. Large herbivore communities dominated by ungulates are common in mainland Africa and provide dung beetles with abundant resources to support diverse communities. In contrast, Madagascar lacks any native ungulates and the most important mammals apart from small mammals are primates (lemurs). As a result, practically all native dung beetle species in Madagascar occur in forests, where most of the lemurs also occur [27,28,33]. It is probably not a coincidence that the timings of Helictopleurina (44–23 Mya) and lemur (44–22 Mya) radiations agree closely [28]. On the other hand, a general feature of dung beetle communities in tropical forests is that a large proportion of the species use carrion rather than dung or use both of these resources [53]. This shift in resource use is thought to be due to fierce resource competition and the generally high rate of decomposition in tropical forests [52,53]. In Madagascar, such an evolutionary shift in resource use is supported by the results for Helictopleurina: the species in the basal clades are generally coprophagous whereas most species in a large derived clade use carrion as their main resource [27]. As a matter of fact, the majority of species in Canthonini and Helictopleurina have a relatively generalized diet, which may explain their success in wet forests in Madagascar.

Apart from the old evolutionary shifts from ungulate to primate dung and from dung to carrion, a more recent ecological shift has been identified in some Malagasy dung beetles in the opposite direction [29,32]. The range of resources available to Malagasy dung beetles has increased in recent times, associated with the arrival of humans to the island 2300 years ago [54]. The most significant change for dung beetles was the introduction of cattle about 1500 years ago. In wet forests, not a single species of dung beetle has shifted to use cattle dung as the primary resource, though there are some feral cattle in many forested regions throughout Madagascar [33]. In contrast, in the dry open areas of western Madagascar the situation is different, with at least 20 cattle dung-using species [32], including both native and introduced species. The resource shift to cattle dung has been studied in more detail in Helictopleurina, in which four *Helictopleurus* species have shifted to cattle dung in open areas [27,29]. Molecular genetic data show that that these four species have expanded their geographical ranges following the shift to the new resource, most probably because of relaxation of interspecific resource competition [29].

## 6. Phylogeographic Structure in Malagasy Dung Beetles

The processes that underpin dung beetle radiations in Madagascar and are responsible for high regional turnover in species composition remain challenges for future research. A start has been made with a small group of four species that belong to the ecologically most successful lineage of dung beetles in Madagascar, the *Nanos* clade, providing insights into the processes responsible for speciation in this group. *Nanos dubitatus*, *N. viettei*, *N. nitens* and *N. “viettei 46”* (new species being described by Olivier Montreuil, [55]) form a monophyletic group (the viettei group, sensu [30]) and have allopatric distributions in eastern Madagascar. *Nanos viettei* is the dominant species in the southern part of the eastern rainforests and is replaced by *N. dubitatus* from central Madagascar northwards, with the transition occurring around the Mangoro river. *Nanos “viettei 46”* is morphologically indistinguishable from *N. viettei*, and has a small geographic range at high altitudes at the northern range limit of *N. viettei. Nanos nitens* is morphologically more distinct from the other species and has a relatively small geographical range in northern Madagascar, with little elevational overlap with *N. dubitatus* [34]. The estimated times of divergence place the split of the *viettei* group from its closest relatives in the early Miocene (23–14 Mya). Divergence within the *viettei* group itself started during the transition between Miocene to Pliocene (7–4 Mya) but further differentiation among the extant taxa started more recently (<2 Mya), coinciding with the glacial cycles of the Pleistocene [30].

What are the processes that best explain the origin and current distribution of genetic variation in this group of species? It seems that initial differentiation consisted of the isolation of northern (*N. dubitatus* and *N. nitens*) and southern (*N. viettei* and *N. “viettei 46”*) lineages. North-south biogeographic division in Madagascar is supported by several studies on many animal groups (e.g. in lemurs, [56]). Further differentiation between and within lineages coincided with the intensification of the glacial cycles during the Pleistocene. It is widely acknowledged that the Quaternary climatic cycles have played an important role in shaping the current distribution of genetic variation in various taxa throughout the globe [57]. In Madagascar, during the glacial cool and dry periods forests have most probably shrunk to smaller areas promoting differentiation of forest-dwelling species in allopatric refugia [1,58,59]. During the warm and humid interglacial periods, forests have been continuous allowing species lineages to extend their ranges. However, competition with congenerics may have prevented species from expanding their ranges widely. Allopatric divergence associated with the Quaternary climatic cycles has been proposed to explain the divergence in many groups of species in Madagascar. These studies have emphasized the role of mountains and watersheds in providing refuge and sourcing the re-colonization during dry and wet cycles, respectively, while major rivers may have constituted barriers to gene flow (e.g., [58,60]). Recent studies have indicated that environmental gradients and diverse climates across Madagascar have played an important role in the generation of local endemism through e.g., parapatric speciation across altitudinal clines [59,61].

**Figure 1:**
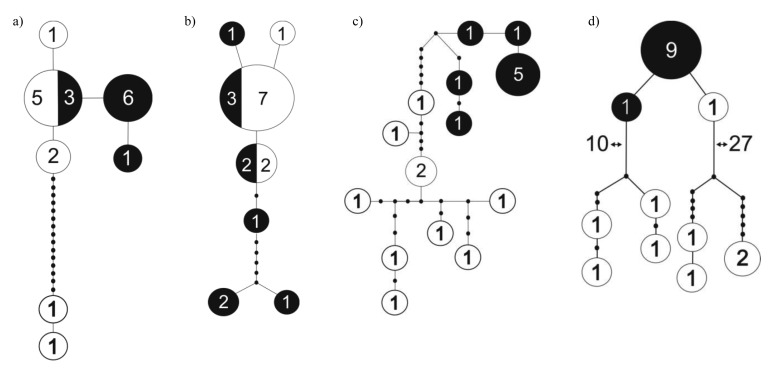
Haplotype networks (mitochondrial COI, 700 bp) for four common species of dung beetles sampled in two nearby (∼40 km) localities in northeastern Madagascar, the Marojejy National Park (represented in white) and the Anjanaharibe-Sud Special Reserve (represented in black). The species are (a) *Helictopleurus obscurus*, (b) *Helictopleurus rudicollis*, (c) *Epilissus emmae* and (d) *Epilissus splendidus*. Numbers inside circles represent the number of individuals with that haplotype. In (d) numbers ‘10’ and ‘27’ represent the number of mutational steps between the adjacent haplotypes. Small black circles represent unsampled or extinct haplotypes.

Knopp *et al.* [31] compared dung beetle species composition and genetic differentiation between populations on the two sides of the Mangoro River, the largest river on the east coast, but found little support for the hypothesis of rivers functioning as dispersal barriers and influencing patterns of microendemism in dung beetles [31]. Another recent study compared levels of genetic differentiation between two nearby (∼40 km) forest localities in northeast Madagascar (Marojejy and Anjanaharibe-Sud) and found contrasting patterns in different species (M. Miinala, unpubl. data). While *Helictopleurus obscurus* and *H. rudicollis* showed little or no genetic differentiation between the localities, *Epilissus emmae* and *E. splendidus* showed marked structure in the distribution of genetic variation with no haplotypes being shared between the two localities (Figure 1). These results suggest that there is substantial genetic differentiation among populations within small spatial scale in some groups of dung beetles but not in others.

All of the different processes mentioned above are likely to have shaped the evolutionary history of dung beetles in Madagascar at some point in time and to a certain extent, but further investigations of genetic differentiation of closely related species are needed to gain better understanding of particular mechanisms. For example, in the *Nanos* clade many species have restricted elevational distributions (unpublished data). A key question is whether speciation has occurred along elevational gradients or whether ancestral species have speciated along the latitude at different altitudinal zones.

### 6.1. The Role of Hybridization and Introgression

The above mentioned studies indicate an important role for genetic divergence in allopatry, perhaps following the fragmentation of previously more continuous ranges during unfavorable climatic periods. In the early stages of the speciation process, gene flow through hybridization is possible in zones of secondary contact, following range expansions during favorable climatic periods, which may slow down the divergence. As an example, although the split between *Nanos dubitatus* and *N. viettei* is old (7–4 Mya), genomic introgression from *N. dubitatus* into *N. viettei* has been detected [30]. The timing estimates suggest that the introgression occurred during the Pleistocene. In general, hybridization occurs mainly between recently differentiated taxa but many taxa retain the capacity to hybridize for long periods after divergence [62]. *Nanos dubitatus* and *N. viettei* possibly still hybridize as they have very similar morphologies and ecologies and have been shown to mate with each other when brought together in the laboratory [34]. A more detailed genetic analysis of the putative secondary contact zone between these two species is necessary to assess the extent of introgression and to elucidate the processes that are keeping these taxa as distinct evolutionary units.

### 6.2. The Role of Ecological Processes

**Figure 2:**
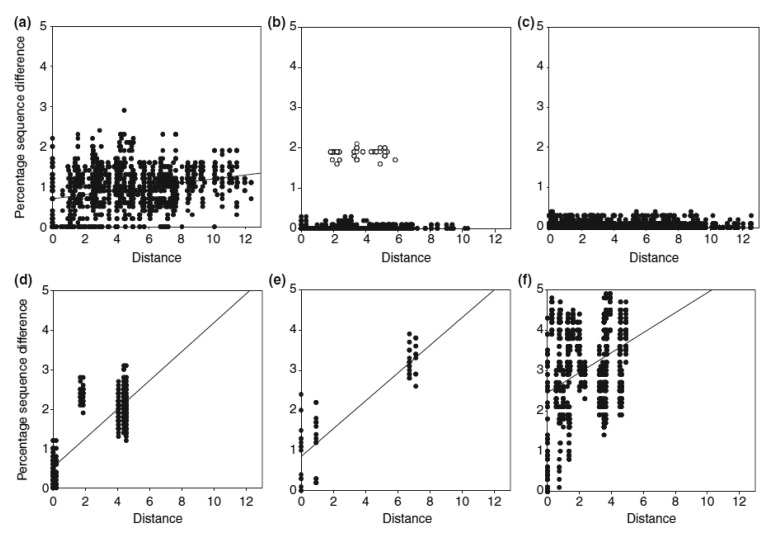
Percentage sequence difference (uncorrected p distances) between pairs of individuals against geographical distance (in degrees, corresponding to 112 km at the equator). Upper row: cattle dung-using species *Helictopleurus quadripunctatus* (a), *Helictopleurus marsyas* (b) and *Helictopleurus neoamplicollis* (c). The open symbols in (b) are for pairs of individuals involving *H. marsyas* and *H. nicollei* [29]. Lower row: forest dwelling species *Helictopleurus unifasciatus* (d), *Helictopleurus perrieri* (e) and *Nanos clypeatus* (f; from Wirta 2008). Figure adapted from Hanski *et al.* [29].

Apart from the complex past evolutionary processes, more contemporary ecological processes can also shape the current distribution of species' genetic diversity. A good example is the recent resource shift to cattle dung documented for four species of *Helictopleurus*, which has left a signature in their spatial genetic variation [29]. Detailed phylogeographic analysis indicated that the shift to cattle dung has occurred within a small geographical region in *Helictopleurus neoamplicollis* and *H. marsyas*, followed by a rapid range expansion resulting in low genetic diversity across their current ranges and no isolation-by-distance pattern (Figure 2). This pattern is in striking contrast with the marked isolation-by-distance pattern in three forest species (the lower row of panels in Figure 2). *Helictopleurus quadripunctatus* exhibits a somewhat intermediate pattern, possibly reflecting a more gradual shift in diet across much of its original range, allowing therefore the maintenance of higher genetic diversity across its current distribution.

## 7. Conclusion: Adaptive *vs.* Non-Adaptive Radiations?

The data that have been accumulated over the past decade and reviewed in this paper suggest that dung beetle radiations in Madagascar have been shaped by several processes. It is clear that during the long history of evolution both geographical and ecological factors have promoted divergence and speciation allowing extensive adaptive and non-adaptive radiations to take place in several lineages, whereas in other lineages speciation has been very limited, and not only because of lack of time. The most recent radiation in Canthonini, involving the *Nanos* clade, has the signatures of a non-adaptive radiation, with species showing allopatric distributions and limited ecological differentiation. This pattern suggests that speciation has occurred in allopatry [28,33]. In contrast, the *Epactoides* clade is a good putative example of adaptive radiation, with pairs of sister species showing dissimilar ecological traits but similar geographical distributions. This pattern suggests that ecological speciation, possibly in sympatry, has contributed to lineage splitting. However, in this case we cannot exclude the possibility of speciation preceding significant ecological differentiation. As stressed by Rundell and Price [63], largely overlapping ranges in sister species that differ in ecological traits could be the result of range expansions following speciation in allopatry. In the subtribe Helictopleurina, species have diverged in terms of body size, habitat selection and food resource use. Many species show largely sympatric distributions with significant ecological differentiation [33], most likely due to ecological adaptation facilitating the co-occurrence of species. However, there are also pairs of sister species in *Helictopleurus* with largely allopatric distributions and similar ecologies, suggesting allopatric speciation with perhaps limited role for ecological mechanisms.

In summary, the several radiations of dung beetles in Madagascar show substantial differences in the distribution of ecological traits and in the extent of spatial overlap in the geographical ranges of related species, suggesting that both adaptive and non-adaptive mechanisms have played a role in their evolution. Despite these differences, all lineages include many microendemic species, highlighting the uniqueness of the biota in Madagascar. Madagascar is both a veritable hotspot of global biodiversity [3] and a fascinating laboratory of evolutionary processes [1].

## Figures and Tables

**Table 1:** Eight independent colonizations of Madagascar by Scarabaeinae dung beetles, with times of subsequent radiations and the number of extant species.

**Tribe/Subtribe**	**Genera**	**Number of Colonizations**	**Start of Divergence (Mya)**	**Number of Extant Species**	**Reference**
**Canthonini**					
	*Arachnodes, Epilissus* and *Apterepilissus*	1	79–49	101	[28]
	*Nanos* and *Apotolamprus*	1	24–15	61	[28]
	*Epactoides*	1	30-19	37	[28]
**Helictopleurina**					
	*Helictopleurus*	1	37–23	66	[27]
**Scarabaeini**					
	*Scarabaeus*	1 or 2	24–15	3	[43]
**Onthophagini**					
	*Onthophagus*	at least 3	unknown	6	[27]
